# Reconstruction of a Giant Wound Induced by Advanced Penile Carcinoma with Bilateral Anteromedial Thigh Flap and Left Deep Circumflex Iliac Artery Flaps

**Published:** 2018-01

**Authors:** Chenyang Ji, Ruiting Li, Ganlin Zhang, Jinming Zhang, Weiqiang Liang, Yuhong Chen

**Affiliations:** Department of Plastic Surgery, Sun Yat-sen Memorial Hosptial, Sun Yat-sen University, Guangzhou, China

**Keywords:** Penile, Squamous cell carcinoma, Abdominoperineal defect, Anteromedial thigh flap, Deep circumflex iliac artery flap

## Abstract

A 39-year-old male patient presented with an extensive putrescent ulceration of abdominoperineal region infiltrated by advanced penile squamous cell carcinoma. To our knowledge, it is the largest defect after aggressive palliative resection of penile squamous cell carcinoma (pSCC) in the literature, which was 36×23 cm. The defect was divided into three sub-defects, and was repaired by bilateral anteromedial thigh (AMT) and left deep circumflex iliac artery (DCIA) flaps. The postoperative course was uneventful and no flap necrosis occurred. The symptom relief was excellent.

## INTRODUCTION

Penile squamous cell carcinoma (pSCC) is a rare disease, making it difficult to establish a standard of care in any of the clinical stages, particularly in advanced disease.^1^ Some patients with advanced pSCC can develop cutaneous metastases involving the inguinal, suprapubic and anterior abdominal wall areas. We report a case of advanced pSCC with an extensive infiltration of abdominoperineal region treated with aggressive palliative resection and reconstruction by bilateral anteromedial thigh (AMT) and left deep circumflex iliac artery (DCIA) flaps.

## CASE REPORT

A 39-year-old patient presented with an extensive putrescent ulceration of abdominoperineal region. The tumor had grown gradually over 2 years. The penectomy and perineal urethrostomy were refused before. Local excision and radiochemotherapy had failed to control. The infiltration was from skin of right anterior superior iliac spine to left anterior superior iliac spine, going across lower abdominal, extending to bilateral inguinal region, and reached 6 cm below left groin. Scrotum was involved. Bilateral inguinal lymph nodes and left pelvic lymph nodes metastasis and invasion of left femoral sheath were observed with MRI. Massive exudation was produced, and hemorrhage was occurred when changing the dressing. 

The operation commenced with aggressive palliative resection by urologist surgeons. The ulcer and area of radiodermatitis with at least 2 cm margins were resected from the layer of deep fascia. Bilateral inguinal lymph node dissection and excision of left femoral sheath were taken. The residual corpora cavernosum of penis was separated and excised. The left spermatic cord and testicle were resected due to tumor invasion. Then, a large skin defect measured 36×23 cm occurred. A partition concept was introduced. The defect was divided into three sub-defects, defined by medioventral line and left arcus cruralis. A combined bilateral AMT and left DCIA flaps were prepared according to preoperative Doppler examination.

**Fig. 1:**
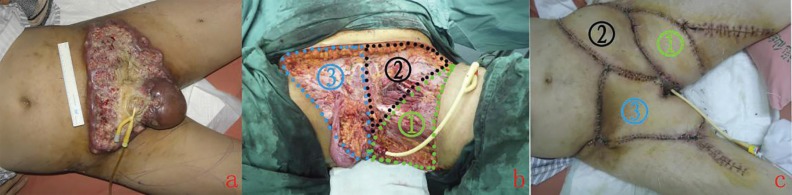
a: Preoperative view. The patient presented with an extensive ulceration of lower abdominal and perineal region infiltrated by advanced penile squamous cell carcinoma.; b: The defect was divided into three sub-defects, defined by medioventral line, left arcus cruralis; c: Three weeks postoperative. The flaps survived well.

A spindle shaped left AMT flap measured 22×10 cm was raised. The distal part was transferred to the lower part of left pudenal thigh, while the proximal end of the flap was sutured at the point of left anterior superior spine. Then, a 27×15 cm spindle shaped left DCIA flap was incised. Musculus obliquus externus abdominis and musculi obliquus internus abdominis were dissected along from the proximal to the middle of the flap, together with the iliac periosteum to ensure the blood supply of the flap. The flap was advanced towards to the bottom of perineal region and sutured to the residual skin of scrotum. At last, a right side triangle AMT flap, which was 30×17 cm, was designed and raised. The medial end of the flap was lift up and the lower end was rotated internally to the bottom of perineal region (Figure 1).

The postoperative course was uneventful and no flap necrosis occurred. Two weeks later, the patient received chemotherapy (paclitaxel liposome+nedaplatin) and endogenic heat therapy, then helical tomotherapy 4 weeks later. However, the tumor metastasis was not controlled. The patient died of electrolyte imbalance and respiratory failure three months later. Local recurrence was observed as cutaneous nodules in the left lliac waist and perineal region with scattered fester.

## DISCUSSION

To reconstruct the defects of pSCC presenting with extensive cutaneous metastasis, a variety flaps have been used, which contained tensor fascia lata flap,^2^ rectus abdominis flap,^3^^,^^4^ gluteus maximus flap,^5^ scrotal advancement flap,^6^ pedicled ALT flap^7^^,^^8^ and VRAM flap.^9^^,^^10^ They have confirmed benefit in patients after radical excision of high-volume inguinal gland metastases and after aggressive palliative resection of extensive cutaneous metastasis. 

Kayes *et al.*^10^ considered that using VRAM flap, even in the very large defects, reliable and adequate wound cover with primary closure of both donor and recipient sites can be achieved. However, the case here showed a giant abdominoperineal defect, the methods mentioned above were presumably unsuitable. The defect accounted for a large proportion of the patient’s body. After planning, we introduced a partition concept, in which. the defect was divided into three subunits, and each subunit was repaired by a single pedicled flap. A combined pedicled bilateral AMT flaps and left DCIA flap were planned.

The AMT flap is an useful alternative to repair the defect of lower abdomen and inguinal region. It was first described by Baek in 1983^11^ as the medial thigh flap and by Song *et al.* in 1984.^12^ In previous reports, the flap was described as a free cutaneous flap, but after recognising the advantages of using it as a pedicle flap for the reconstruction of the lower abdomen, perineal area, the groin and donor site of ALT flap,^13^^-^^15^ it was soon popular as a pedicle flap. 

The DCIA flap, usually regarded as a free flap, can be used in maxillofacial surgery because of its capability of carrying iliac bone. The studies of perforators^16^ and terminal branch^17^ have facilitated the usage of DCIA flap as a pedicled flap in covering the defect of lower abdomen, inguinal region and perineal region. Ramasastry *et al.*^18^ had used the internal oblique muscle as a transpositional flap based on the ascending branch of the DCIA, for coverage of a groin defect with exposed femoral vessels.

The partition concept in this case has advantages. The primary defect was divided into several subunits, therefore the areas to be repaired became relatively small, which ensured the operability. The blood circulation disturbance in a certain one large single flap could be prevented. Meanwhile, the multiple pedicled flaps avoided complications of free flap.^19^^-^^21^ What’s more each smaller secondary defect can be closed easily without skin grafting or “buddy flap”^22^ for the donor-site defect. 

To our knowledge, it is the largest defect after aggressive palliative resection of pSCC in the literature so far. The defect was reconstructed with bilateral AMT flaps and left DCIA flap, which also is the first reported. It is a difficult patient to treat but we have shown that an aggressive surgical approach can alleviate the symptoms associated with cutaneous disease and avoid potentially disastrous outcomes (i.e. exsanguination).
